# Effect of Acute Illness on Contact Patterns, Malawi, 2017

**DOI:** 10.3201/eid2601.181539

**Published:** 2020-01

**Authors:** Judith R. Glynn, Estelle McLean, Jullita Malava, Albert Dube, Cynthia Katundu, Amelia C. Crampin, Steffen Geis

**Affiliations:** London School of Hygiene & Tropical Medicine, London, UK (J.R. Glynn, E. McLean, A.C. Crampin, S. Geis);; Malawi Epidemiology and Intervention Research Unit, Chilumba, Malawi (E. McLean, J. Malava, A. Dube, C. Katunda, A.C. Crampin, S. Geis)

**Keywords:** Infection control, infection transmission, Malawi, Africa, contact patterns, acute illness

## Abstract

The way persons interact when ill could profoundly affect transmission of infectious agents. To obtain data on these patterns in Africa, we recorded self-reported named contacts and opportunities for casual contact in rural northern Malawi. We interviewed 384 patients and 257 caregivers about contacts over three 24-hour periods: day of the clinic visit for acute illness, the next day, and 2 weeks later when well. For participants of all ages, the number of adult contacts and the proportion using public transportation was higher on the day of the clinic visit than later when well. Compared with the day after the clinic visit, well participants (2 weeks later) named a mean of 0.4 extra contacts; the increase was larger for indoor or prolonged contacts. When well, participants were more likely to visit other houses and congregate settings. When ill, they had more visitors at home. These findings could help refine models of infection spread.

Knowledge of patterns of contact between persons is central to understanding transmission of infections and for designing control strategies (*1*–*3*). Until recently, models estimating the spread of infections assumed random mixing, which is too simplistic, or used data from studies in high-income countries, which may not be relevant for low-income settings (*4*–*7*). Although a few studies have been conducted in low-income countries, including 4 published studies from Africa (*8*–*11*), none have assessed how contact patterns change after illness. Studies have shown that persons predominantly mix with those of the same age (assortative mixing) (*11*,*12*), but the degree of intergenerational mixing and numbers of contacts vary between settings, depending on population characteristics such as household size and structure, income-generating activities, and population density (*9*,*12*).

Almost all studies of contact patterns have involved healthy persons, but infection spread will be greatly influenced by the way persons mix when they are ill (*13*). Illness probably affects the contacts and movements of the sick persons and their household members. These altered patterns will be a key determinant of infection spread but are largely unknown even in high-income settings (*13*,*14*). A study of the effect of influenza-like symptoms in the United Kingdom indicated that changes in contact patterns after illness resulted in a reduced reproduction number (average number of cases generated by 1 case-patient) to <30% of the value it would have had if contact patterns had not changed (*13*). This effect would dramatically alter spread of infection in the population. To help learn whether similar changes occur in populations in Africa, we studied contact patterns during and after illness in a rural area in Karonga District, northern Malawi.

## Methods

The study was conducted within a demographic surveillance area in rural northern Malawi (*15*). Our previous pilot work showed that keeping a diary and being interviewed about contacts in the previous 24 hours are acceptable to the population but that when each is done independently, both fail to include all contacts. We therefore combined these methods; participants and interviewers could refer to the diary (Appendix 1) as a memory aid during the interview, and interviewers were instructed to probe carefully for contacts. Contacts were defined as persons with whom the participant had face-to-face conversations or skin contact, not persons they simply passed and greeted.

Project staff based at the clinic recruited participants from among clinic patients with symptoms suggestive of acute infectious disease (e.g., fever, respiratory symptoms, diarrhea, vomiting). After obtaining written consent, an interviewer explained to the patient, caregiver, or both how to keep a diary of contacts, starting with the day of the clinic visit. 

On the day after the clinic visit, the interviewer visited the home to ask about activities of the previous day and contacts made (number, age, sex, duration, context), who was living in the household at that time and socioeconomic variables, and any movement outside the household in the past 24 hours (including visits to other households; use of taxis/minibuses; and visits to congregate settings such as churches, markets, funerals, school). This visit was also an opportunity to address any difficulties the participants encountered with regard to keeping the diary. The participant was given a new diary to use over the next 24 hours, and another visit and interview were scheduled for the next day. For children <18 years of age, mothers/guardians who attended the clinic with them helped the children keep the diary and were also asked to keep a diary for themselves for the same periods, to assess contacts while caregiving. We collected data for 2 days of illness because the day of the clinic visit may be atypical and because explaining the diary at the clinic while the patient is ill and wanting to get home cannot take very long, so a refresher session may be necessary.

Two weeks later, each participant was revisited, given a diary, and interviewed the next day to collect information about a 24-hour period while well. If possible, these visits were on the same day of the week as the first home visit and conducted by the same interviewer. If the patient was still sick, the visit was rescheduled.

Sample size calculations for this type of study are not well defined. Previous studies have recorded data for <300 to >1,000 persons. We planned to recruit 400 patients stratified by sex and by age (0–5, 6–17, 18–49, >50 years); however, because few adult men and few older adults attended the clinic, we combined the adult age groups. In addition, we recruited caregivers for participants <18 years of age.

Analyses describe contact patterns of participants in the different groups (patient and caregiver, different visits) by using means and differences in mean numbers of contacts. The primary comparison was between when ill, the day after the clinic visit, and when well (2 weeks later) for patients and caregivers, restricted to those visited on the same day of the week for the 2 visits. To assess changes in visits to congregate settings, the mean difference in proportions attending was calculated by scoring a visit as 1 and no visit as 0. For persons with unchanged patterns when ill and well (either visiting or not visiting a congregate setting on both occasions) the difference would be 0; for those visiting when well but not when ill, the difference would be 1; and for those visiting when ill but not when well, the difference would be –1. Assuming a normal distribution of the differences, we calculated exact 95% CIs. The study was approved by the National Health Sciences Research Committee, Malawi (no. 1695) and by the ethics committee of the London School of Hygiene & Tropical Medicine, UK (no. 12023).

## Results

At the end of the recruitment period, we had interviewed 384 patients and 257 caregivers. A total of 343 patients and 233 caregivers completed interviews for all 3 visits; 18 patients refused and 41 consented but did not appear for the initial interview in the clinic. 

The population was rural; 71% (259/366) of adults were farmers and 35% (129/366) of adults had not completed primary school (Table 1). Most participants had regular contact (at least monthly) with animals. Although most participants (84%, 515/616) traveled outside their village at least once a month, only 13% (79/616) had ever left the district. Almost all patients had fever; 35% (130/369) had respiratory symptoms; and 20% (74/369) had diarrhea, vomiting, or both

**Table 1:** Characteristics of participants in study of the effect of acute illness on contact patterns, Malawi, 2017

Characteristic	No. (%)
Patient age, y/sex	
0–5/F	87 (23.6)
0–5/M	79 (21.4)
6–1/F	47 (12.7)
6–17/M	37 (10.0)
>18/F	88 (23.8)
>18/M	31 (8.4)
Caregiver	
F	207 (83.8)
M	40 (16.2)
Initial symptoms of patients	
Fever	364 (98.6)
Respiratory symptoms	130 (35.2)
Diarrhea/vomiting	74 (20.1)
Patients and caregivers	
Schooling (adults)	
<Primary	129 (35.2)
Primary	198 (54.1)
Secondary (completed)	39 (10.7)
Occupation of adults	
Farmer	259 (70.8)
Skilled nonmanual	51 (13.9)
Other	56 (15.3)
Travel outside village	
Most days	231 (37.5)
>1×/wk	174 (28.2)
> 1×/mo	110 (17.9)
<1×/mo	41 (6.7)
Never	59 (9.6)
Ever travel outside	
District	79 (12.8)
Region	54 (8.8)
Country	6 (1.0)
Animal contact (≥monthly)	
Ducks/chickens	375 (60.9)
Pigs	137 (22.2)
Animal feces	336 (54.5)

**Figure 1:**
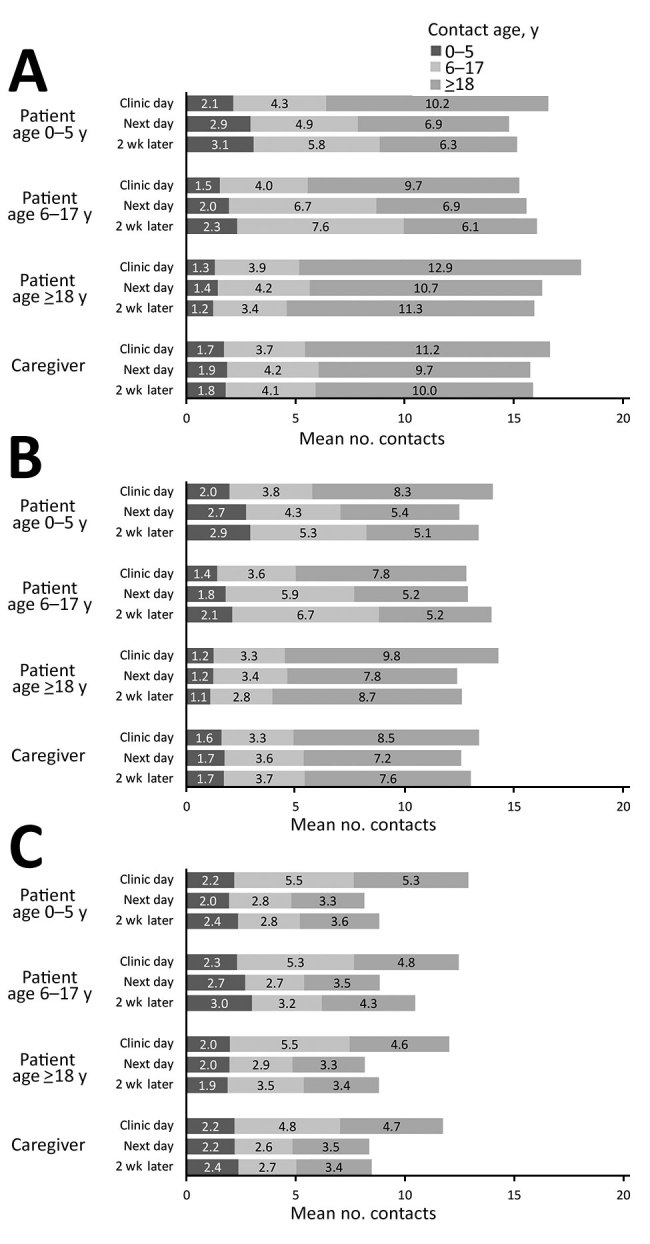
Contact patterns, by age of study participant, age of contact, and visit, in study of the effect of acute illness on contact patterns, Malawi, 2017. Mean number of close contacts per 24-hour period overall (A); restricted to contacts of >10 minutes (B); restricted to indoor contacts (C).

Patients had a mean (± SD) of 16.8 (± 5.6) contacts on the day of the clinic visit, 15.4 (± 5.9) the next day, and 15.6 (± 5.5) 2 weeks later when well (Figure 1; Appendix 2 Figure 1). Mean contact numbers for caregivers were similar to those for patients for the three 24-hour periods: 16.7 (± 5.2), 15.7 (± 5.6), and 15.9 (± 5.8). When analysis was restricted to contacts involving touch, the mean numbers of contacts were 10.6 (± 4.6), 9.3 (± 4.8), and 10.1 (± 5.2) for patients and 9.6 (± 4.6), 9.0 (± 4.3), and 9.3 (± 4.8) for caregivers for each of the 3 periods. When analysis was restricted to contacts of at least 10 minutes’ duration, the mean numbers of contacts were 14.3 (± 5.0), 13.0 (± 5.1), and 13.6 (± 5.1) for patients and 14.0 (± 4.7), 13.2 (± 4.7), and 13.6 (± 5.2) for caregivers. Among patients with respiratory symptoms, the mean numbers of contacts were 16.6 (± 5.4), 15.4 (± 6.3), and 15.9 (± 5.8) for the 3 periods; among those with diarrhea/vomiting, mean contact numbers were 15.2 (± 4.8), 14.1 (± 5.6), and 14.8 (± 6.2).

**Figure 2:**
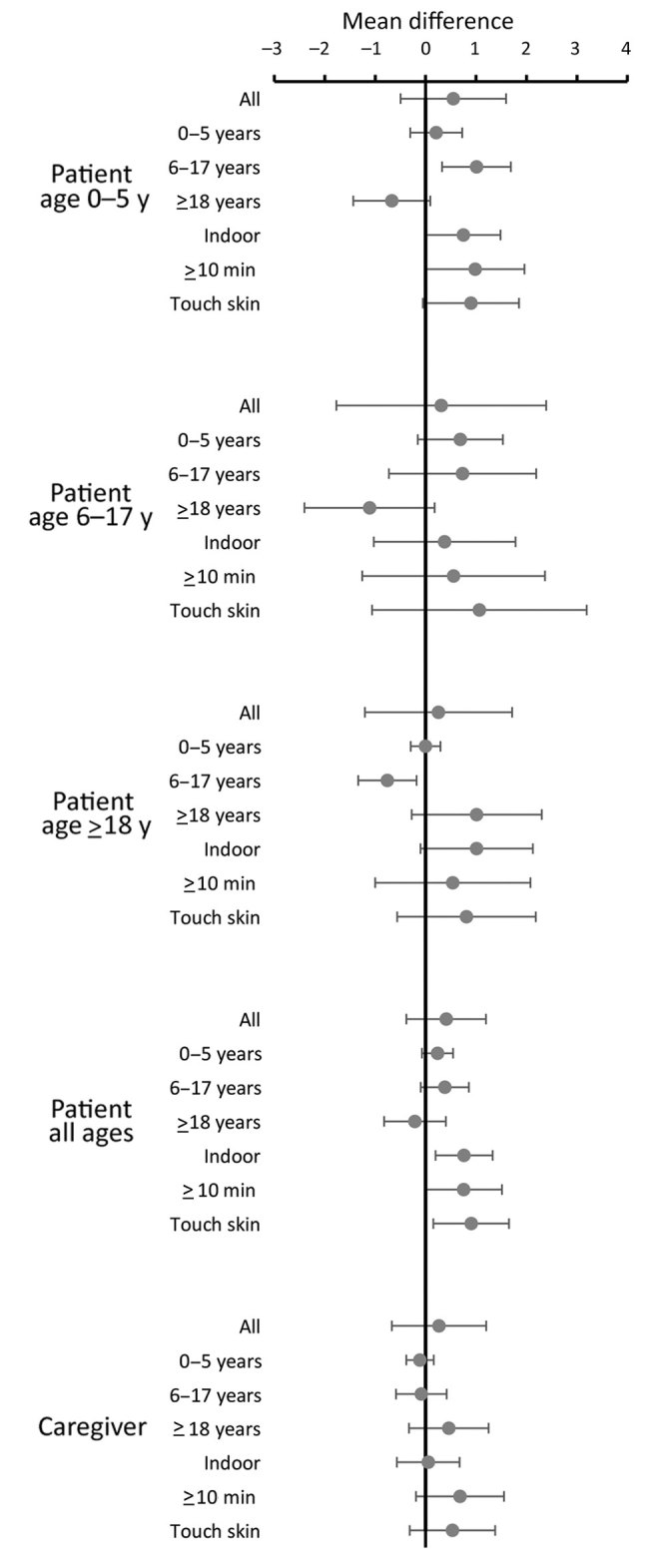
Mean differences in numbers of contacts for study participants when well compared with when ill (the day after the clinic visit), restricted to persons seen on the same day of the week when well and when ill, in study of the effect of acute illness on contact patterns, Malawi, 2017. Characteristics in second column refer to contacts. Mean difference >0 implies more contacts when well, mean difference <0 implies more contacts when ill. Error bars indicate 95% CIs.

Overall, the contact patterns while ill on the day after the clinic visit were similar to those after recovery in terms of number and age pattern of contacts (Figure 1, panel A; Appendix 2 Figure 2). Contact numbers were similar for men and women, and because relatively few adult men were interviewed, we combined the results. We found some evidence of assortative mixing (like with like) for age. Attending the clinic led to more adult contacts for all participants and fewer child contacts for children.

When we restricted analysis to contacts of at least 10 minutes’ duration, we found 2–3 fewer contacts per person but an overall similar pattern (Figure 1, panel B). Participants had more indoor contacts on the day of the clinic visit, but this number differed little on the other days (Figure 1, panel C).

We found more differences in the type of contact. On the day of the clinic visit, patients met an average of 3.2 contacts they had never met before and caregivers an average of 2.8. On the next day, these numbers were 0.8 and 0.9; 2 weeks later, they were 0.5 and 0.6.

Contact patterns can vary according to day of the week. Further analysis of change of contact patterns compared the day after the clinic visit with 2 weeks later when patients were well for persons who were interviewed on the same day of the week for these 2 periods (228 patients and 154 caregivers). We found no large differences in contact numbers. Overall, patients had a mean (95% CI) of 0.4 (−0.4 to 1.2) extra contacts when well, an increase of 0.8 (0.2–1.3) for indoor contacts, 0.8 (0–1.5) for contacts of >10 minutes, and 0.9 (0.2–1.7) for contacts involving touch. Children tended to have more contacts with children and fewer with adults when well, and adults tended to have more contacts with adults and fewer with children when well, but all differences were small (<1 contact/day) (Figure 2).

**Figure 3:**
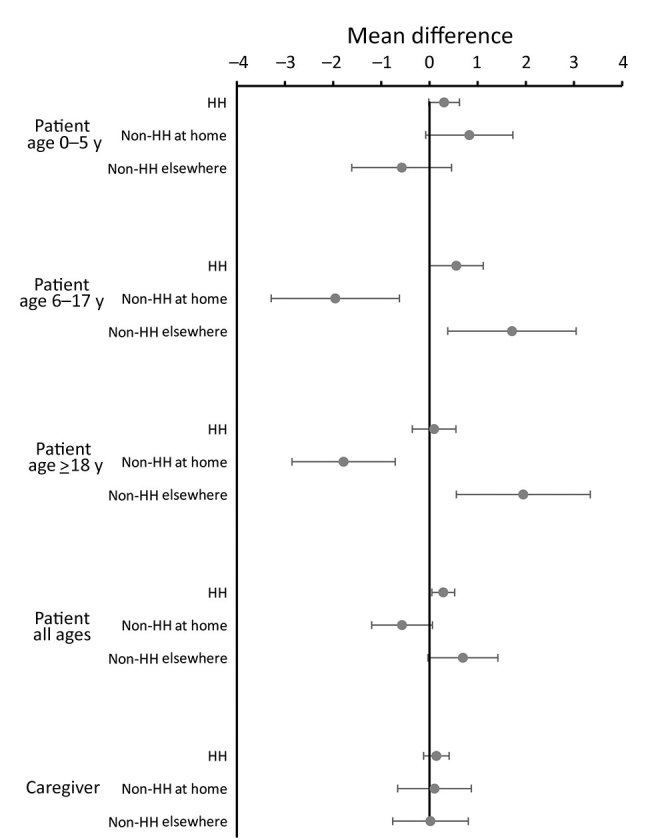
Mean differences in numbers of HH member contacts and non-HH members seen as contacts at the home and elsewhere by study participants when well compared with when ill (the day after the clinic visit), restricted to persons seen on the same day of the week when well and when ill, in study of the effect of acute illness on contact patterns, Malawi, 2017. Mean difference >0 implies more contacts when well; mean difference <0 implies more contacts when ill. Error bars indicate 95% CIs. HH, household.

Household contacts changed little for participants in all age groups (Figure 3; Appendix 2 Figure 3). While well, children 6–17 years of age and adults had an average of 2 fewer non–household contacts at home and 2 more nonhousehold contacts outside the home. We found little difference in household contacts for the youngest children or caregivers. 

**Figure 4:**
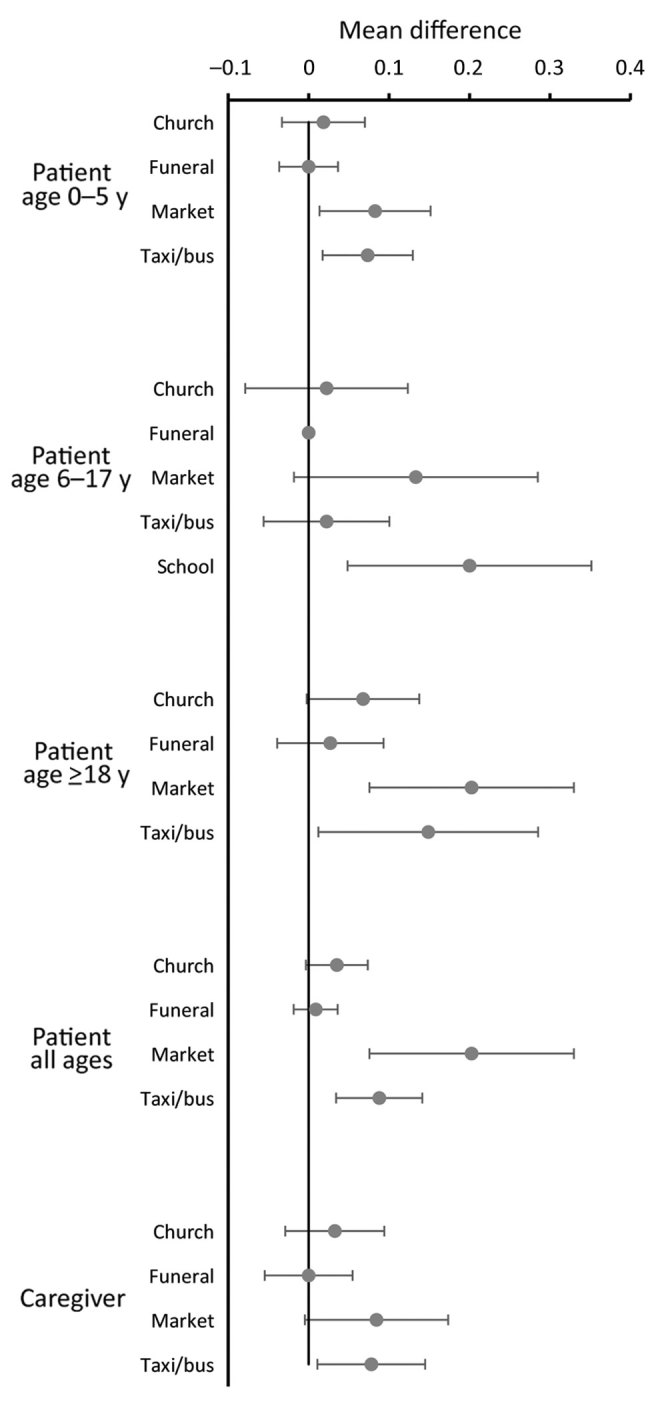
Mean differences in proportions of persons attending congregate settings when well compared with when ill (the day after the clinic visit), restricted to persons seen on the same day of the week when well and when ill, in study of the effect of acute illness on contact patterns, Malawi, 2017. Mean difference >0 implies more visits when well; mean difference <0 implies more visits when ill. Error bars indicate 95% CIs.

**Figure 5:**
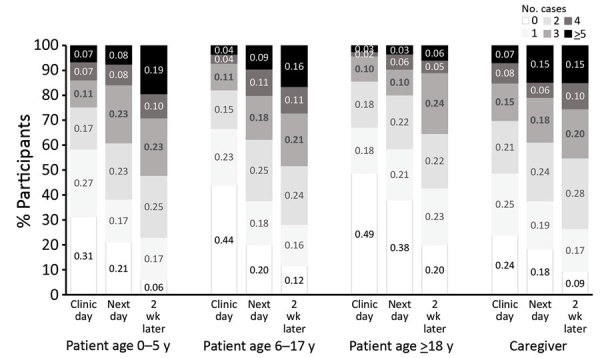
Proportion of all participants visiting 0, 1, 2, 3, 4, or >5 households in each 24-hour period during study of effects of acute illness on contact patterns, Malawi, 2017.

In addition to the individual contacts, we asked about congregate and other settings. Patients were more likely to visit churches, funerals, markets, school, and to travel by public transportation (taxi/bus) when well than on the day after the clinic visit (Table 2). For caregivers, the differences in these visits were smaller. We also compared the difference in these visits between the day after the clinic visit and when well for those interviewed on the same day of the week (Figure 4). When well, participants of all ages were more likely to visit the market, adults and preschool-age children were more likely to use public transportation, adults were more likely to attend church, school-age children were more likely to go to school, and participants of all groups were more likely to visit other households and to visit more such households (Figure 5). We found no differences in funeral attendance, but the numbers were small.

**Table 2:** Number and proportions of patients and caregivers who visited congregate settings in 24-hour periods in study of the effect of acute illness on contact patterns, Malawi, 2017

Setting, date of visit	Patients, no. (%)			Caregivers
	Age 0–5, y	Age 6–17, y	Age >18 y	
All*				
Clinic day	166	84	119	247
Next day	169	85	116	250
2 wk later	165	85	119	248
Church				
Clinic day	1 (0.6)	3 (3.6)	2 (1.7)	11 (4.5)
Next day	11 (6.5)	4 (4.7)	5 (4.3)	23 (9.2)
2 wk later	13 (7.9)	10 (11.8)	11 (9.2)	32 (12.9)
Funeral				
Clinic day	2 (1.2)	0	1 (0.8)	6 (2.4)
Next day	5 (3.0)	0	2 (1.7)	15 (6.0)
2 wk later	4 (2.4)	0	8 (6.7)	14 (5.6)
Market				
Clinic day	45 (27.1)	16 (19.0)	28 (23.5)	86 (34.8)
Next day	14 (8.3)	8 (9.4)	28 (24.1)	68 (27.2)
2 wk later	26 (15.8)	22 (25.9)	52 (43.7)	92 (37.1)
Vehicle				
Clinic day	107 (64.5)	50 (59.5)	65 (54.6)	153 (61.9)
Next day	5 (3.0)	4 (4.7)	15 (12.9)	27 (10.8)
2 wk later	15 (9.1)	4 (4.7)	30 (25.2)	33 (13.3)
School				
Clinic day	1 (0.6)	2 (2.4)	1 (0.8)	3 (1.2)
Next day	3 (1.8)	10 (11.8)	6 (5.2)	3 (1.2)
2 wk later	12 (7.3)	26 (30.6)	7 (5.9)	5 (2.0)
Any other households				
Clinic day	114 (68.7)	47 (56.0)	61 (51.3)	187 (76.1)
Next day	142 (84.0)	68 (80.0)	72 (62.1)	204 (81.6)
2 wk later	155 (93.9)	75 (88.2)	95 (79.8)	225 (90.7)

*Numbers vary between visits because a few persons missed interviews.

## Discussion

In this study of the effect of illness on human contact patterns in rural Malawi, we found a slight reduction in named contacts when ill (the day after the clinic visit) than when well (2 weeks later) and a larger reduction in attendance at congregate settings and visits to other households when ill. Also when ill, on the day of the clinic visit the number of contacts was slightly higher than on the other days, and vehicle use was greatly increased. We also showed similar changes in contact patterns of caregivers.

The small change in named contacts between the day after the clinic visit and when well 2 weeks later can partly be explained by many of the contacts being members of the same household and by the reduction in contacts outside the house when ill being offset by visitors to the house (Figure 3). However, given the greater change in the number of households visited and the opportunities for meeting people at congregate settings, the small difference might also result from the difficulties with collecting accurate contact information. We used the combination of a diary as a memory aid and probing by interviewers. The probing often revealed more contacts than initially recorded. It also enabled discussion with the interviewer about who should be included, which should have helped consistency. Even with this help, however, accurately recording contacts is difficult, especially in a setting of low literacy, and we found some evidence of a tendency to stop at 15 contacts (Appendix 2 Figure 1), which corresponds with the end of the first page of the interviewers’ sheets.

Our results from the participants when well are in the range of findings elsewhere in Africa, but definitions have varied, making direct comparison difficult. In Kenya, participants reported a mean of 17.7 contacts per day involving touch (*8*); in communities in Zambia and South Africa, adult participants reported a mean of 4.9 close contacts (shared conversation longer than a greeting) and 10.4 casual contacts (shared indoor space) per day (*9*); and in a township in South Africa, participants reported a median of 20 close contacts per day, according to a definition similar to ours (*10*). Results from studies in Europe that used a definition similar to ours ranged from 8 to 20 contacts per day (*12*). Some of the variation probably reflects the different settings, and some probably reflects the methods.

 As elsewhere, we found evidence of assortative age mixing (*8*,*10*,*11*), which was reduced when persons were ill, as has been found in the United Kingdom (*13*). The direct comparison of contact patterns for the same persons when well and when ill effectively controls for possible confounding (e.g., by age, sex, or socioeconomic status). It also controls for individual variation in ability to remember contacts. Although it is possible that the higher contact numbers seen on the third visit reflect improved learning and recall, or less distraction by illness, the change in the pattern of types of contact (Figures 2, 3) suggests that these are not the explanation.

The change in households visited and congregate settings attended were more striking than the changes in named contacts. These settings include those that may play a role in infection transmission, such as churches and public transportation, where overcrowding is common, ventilation often poor, and the number of casual contacts can be large (*16*,*17*). Healthcare centers are well recognized as places where infection spread is likely, and to get to the healthcare center, most patients used public transportation. On the day after the clinic visit, congregate setting attendance and household visiting were lower than 2 weeks later when well. The lower market attendance on the day after the clinic visit may partly result from having combined a visit to the market with the clinic visit. Of note, visiting other households was very common for persons of all ages, even when ill (Figure 5).

Our results quantify the changes in contact patterns when persons are ill in rural Africa. Although visiting the clinic increased contacts and being ill decreased contacts, changes in named contacts were small (a reduction in outside contacts was offset by visitors at home). For many infections, the changes in casual contact that were seen would probably have more effect on transmission than the smaller changes in named contacts. These findings could be used to refine models of infection spread.

Appendix 1 Pictorial diary given to participants in study of effects of acute illness on contact patterns, Malawi, 2017.

Appendix 2 Supplemental data from study of effects of acute illness on contact patterns, Malawi, 2017.
