# Supplementary material for: Effect of Acute Illness on Contact Patterns, Malawi, 2017
Source: Emerg Infect Dis. 2020 Jan;26(1):44–50. doi: 10.3201/eid2601.181539 (PMC6924881; doi:10.3201/eid2601.181539)
Supplement: Appendix 1 — Pictorial diary given to participants in study of effects of acute illness on contact patterns, Malawi, 2017. [file 18-1539-Techapp-s1.pdf]

Article DOI: https://doi.org/10.3201/eid2601.181539

Effects of Acute Illness on Contact Patterns, Malawi, 2017

Appendix 1

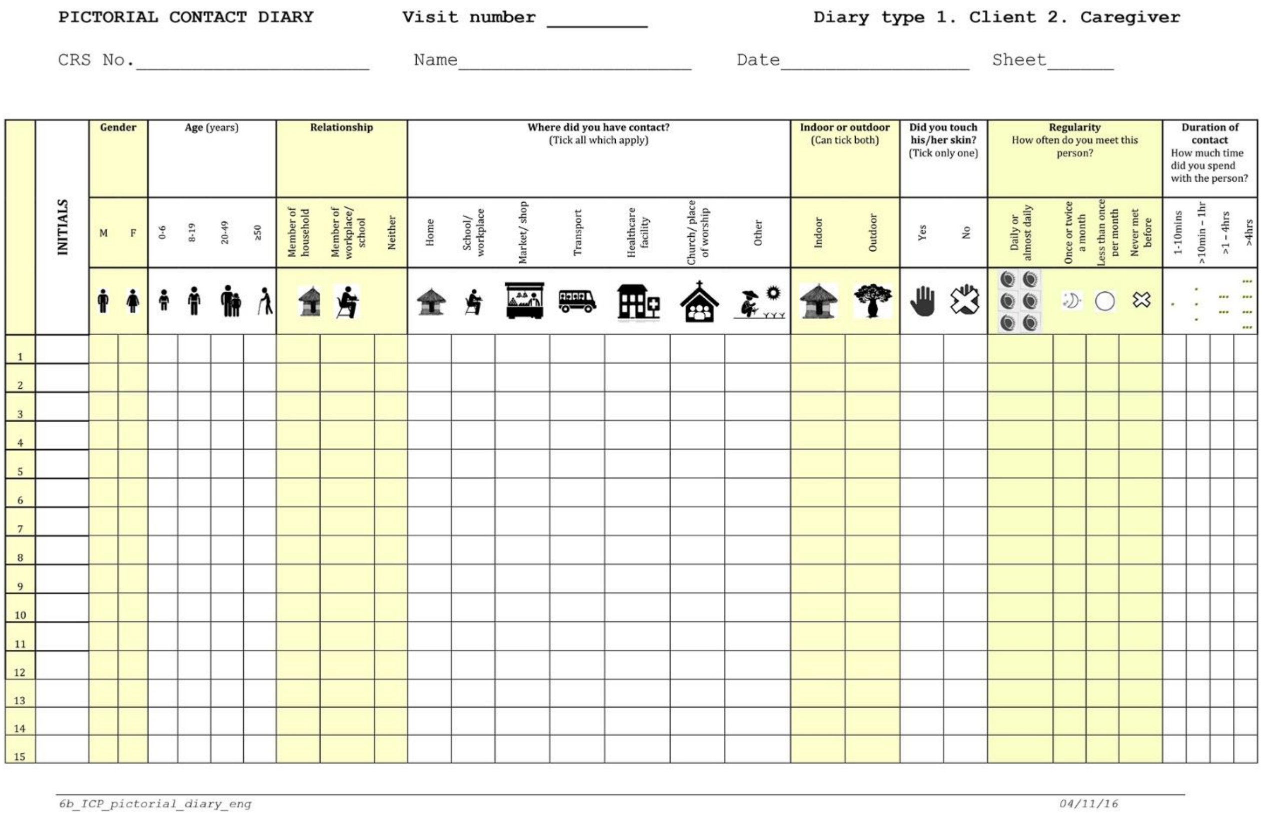

PICTORIAL CONTACT DIARY Visit number ________ Diary type 1. Client 2. Caregiver

CRS No.____________________ Name____________________ Date________________ Sheet______

		Gender		Age (years)				Relationship			Where did you have contact? (Tick all which apply)							Indoor or outdoor (Can tick both)		Did you touch his/her skin? (Tick only one)		Regularity How often do you meet this person?				Duration of contact How much time did you spend with the person?			
	INITIALS	M	F	0-6	8-19	20-49	≥50	Member of household	Member of workplace/ school	Neither	Home	School/ workplace	Market/ shop	Transport	Healthcare facility	Church/ place of worship	Other	Indoor	Outdoor	Yes	No	Daily or almost daily	Once or twice a month	Less than once per month	Never met before	1-10mins	>10min – 1hr	>1 – 4hrs	>4hrs
1																													
2																													
3																													
4																													
5																													
6																													
7																													
8																													
9																													
10																													
11																													
12																													
13																													
14																													
15																													

6b_ICP_pictorial_diary_eng 04/11/16
