# Supplementary material for: Effect of Acute Illness on Contact Patterns, Malawi, 2017
Source: Emerg Infect Dis. 2020 Jan;26(1):44–50. doi: 10.3201/eid2601.181539 (PMC6924881; doi:10.3201/eid2601.181539)
Supplement: Appendix 2 — Supplemental data from study of effects of acute illness on contact patterns, Malawi, 2017. [file 18-1539-Techapp-s2.pdf]

Article DOI: https://doi.org/10.3201/eid2601.181539

# Effects of Acute Illness on Contact Patterns, Malawi, 2017

## Appendix 2

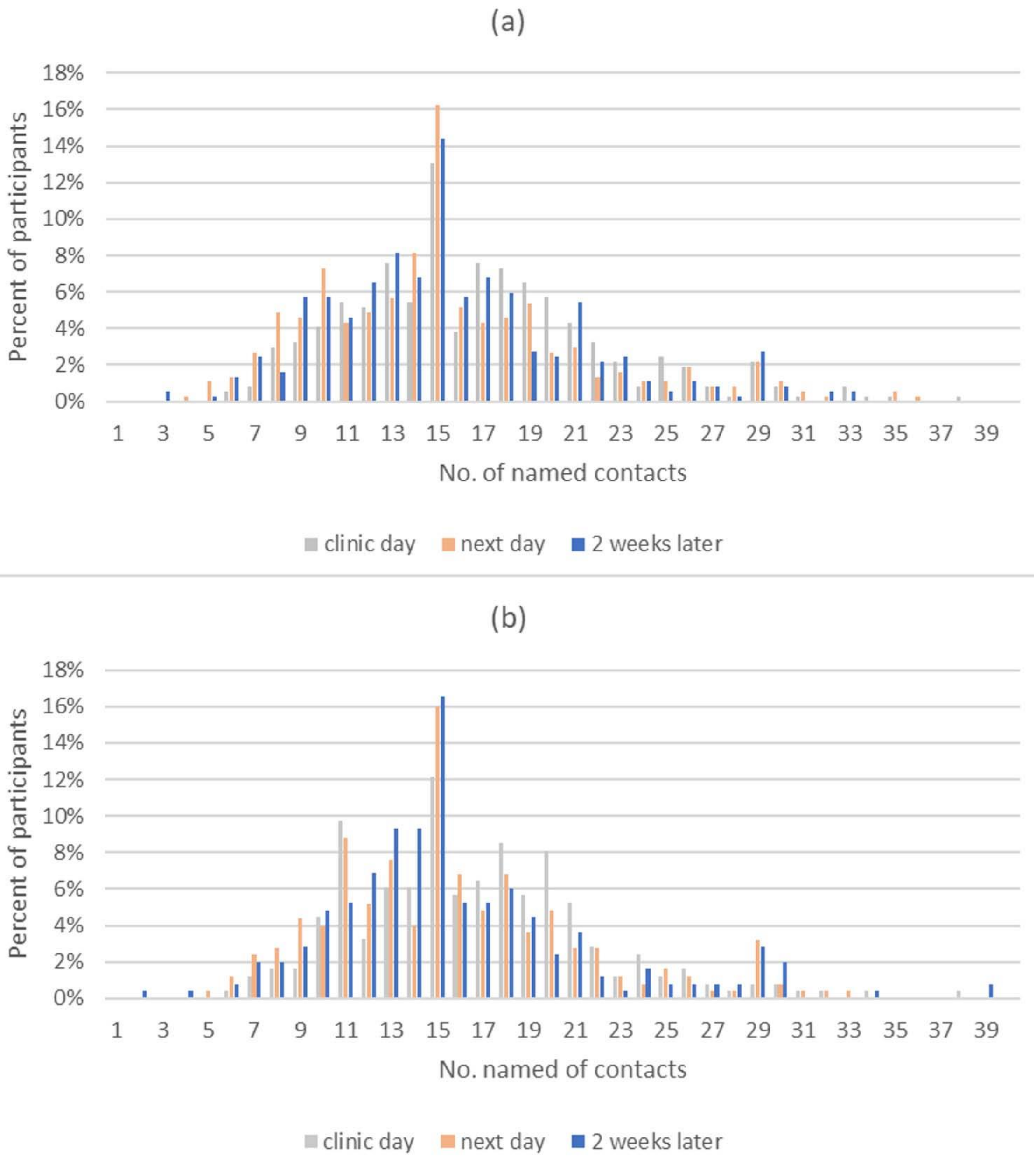


**Figure 1**. Frequency distribution of number of contacts in the three 24-hour periods of (a) patients and (b) caregivers.

## ALL CONTACTS

| | | | All | | | | Duration >=10min | | | | Indoor | | | |
|---|---|---|---|---|---|---|---|---|---|---|---|---|---|---|
| | | N | All | 0-5y | 6-17y | 18+y | All | 0-5y | 6-17y | 18+y | All | 0-5y | 6-17y | 18+y |
| Ill | Patient | 228 | 15.5 | 2.2 | 5.0 | 8.2 | 13.1 | 2.0 | 4.3 | 6.3 | 6.4 | 0.9 | 2.2 | 3.4 |
| | 0-5y | 109 | 14.6 | 3.0 | 4.8 | 6.8 | 12.8 | 2.8 | 4.3 | 5.4 | 6.0 | 0.9 | 2.0 | 3.1 |
| | 6-17y | 45 | 15.9 | 1.8 | 6.9 | 7.2 | 13.6 | 1.7 | 6.0 | 5.3 | 7.2 | 0.9 | 2.9 | 3.4 |
| | 18+y | 74 | 16.4 | 1.3 | 4.2 | 11.0 | 13.2 | 1.1 | 3.4 | 8.4 | 6.5 | 0.9 | 2.0 | 3.6 |
| | Caregiver | 154 | 15.6 | 2.0 | 4.2 | 9.4 | 13.0 | 1.8 | 3.6 | 7.1 | 6.6 | 1.3 | 2.2 | 3.0 |
| Well | Patient | 228 | 15.9 | 2.5 | 5.4 | 8.0 | 13.9 | 2.3 | 4.8 | 6.5 | 7.2 | 1.1 | 2.4 | 3.7 |
| | 0-5y | 109 | 15.2 | 3.3 | 5.8 | 6.1 | 13.8 | 3.1 | 5.4 | 5.1 | 6.8 | 1.2 | 2.4 | 3.2 |
| | 6-17y | 45 | 16.2 | 2.5 | 7.7 | 6.0 | 14.1 | 2.1 | 6.6 | 5.1 | 7.6 | 1.1 | 2.9 | 3.6 |
| | 18+y | 74 | 16.7 | 1.3 | 3.4 | 12.0 | 13.8 | 1.1 | 2.8 | 9.4 | 7.5 | 0.9 | 1.9 | 4.6 |
| | Caregiver | 154 | 15.9 | 1.8 | 4.1 | 9.9 | 13.7 | 1.8 | 3.8 | 7.6 | 6.6 | 1.3 | 2.3 | 3.0 |

## HOUSEHOLD CONTACTS

| | | | All | | | | Duration >=10min | | | | Indoor | | | |
|---|---|---|---|---|---|---|---|---|---|---|---|---|---|---|
| | | N | All | 0-5y | 6-17y | 18+y | All | 0-5y | 6-17y | 18+y | All | 0-5y | 6-17y | 18+y |
| Ill | Patient | 228 | 4.4 | 0.6 | 1.6 | 2.2 | 4.2 | 0.6 | 1.6 | 2.1 | 4.2 | 0.6 | 1.5 | 2.1 |
| | 0-5y | 109 | 4.4 | 0.5 | 1.6 | 2.3 | 4.2 | 0.5 | 1.5 | 2.2 | 4.2 | 0.5 | 1.5 | 2.2 |
| | 6-17y | 45 | 4.6 | 0.6 | 1.9 | 2.2 | 4.6 | 0.6 | 1.8 | 2.2 | 4.6 | 0.6 | 1.8 | 2.2 |
| | 18+y | 74 | 4.2 | 0.7 | 1.5 | 2.0 | 4.0 | 0.7 | 1.4 | 1.9 | 3.9 | 0.7 | 1.5 | 1.8 |
| | Caregiver | 154 | 4.4 | 1.1 | 1.8 | 1.5 | 4.3 | 1.1 | 1.8 | 1.4 | 4.2 | 1.1 | 1.7 | 1.4 |
| Well | Patient | 228 | 4.6 | 0.7 | 1.7 | 2.3 | 4.6 | 0.7 | 1.7 | 2.3 | 4.6 | 0.7 | 1.6 | 2.3 |
| | 0-5y | 109 | 4.7 | 0.6 | 1.7 | 2.3 | 4.6 | 0.6 | 1.7 | 2.3 | 4.5 | 0.6 | 1.7 | 2.3 |
| | 6-17y | 45 | 5.2 | 0.7 | 1.8 | 2.7 | 5.1 | 0.7 | 1.7 | 2.6 | 5.2 | 0.7 | 1.7 | 2.7 |
| | 18+y | 74 | 4.3 | 0.7 | 1.6 | 2.0 | 4.3 | 0.7 | 1.6 | 2.0 | 4.2 | 0.7 | 1.5 | 2.0 |
| | Caregiver | 154 | 4.5 | 1.2 | 1.8 | 1.6 | 4.5 | 1.2 | 1.8 | 1.5 | 4.4 | 1.2 | 1.8 | 1.5 |

## NON-HOUSEHOLD CONTACTS

| | | | All | | | | Duration >=10min | | | | Indoor | | | |
|---|---|---|---|---|---|---|---|---|---|---|---|---|---|---|
| | | N | All | 0-5y | 6-17y | 18+y | All | 0-5y | 6-17y | 18+y | All | 0-5y | 6-17y | 18+y |
| Ill | Patient | 228 | 11.1 | 1.6 | 3.4 | 6.1 | 8.9 | 1.5 | 2.8 | 4.2 | 2.2 | 0.3 | 0.6 | 1.3 |
| | 0-5y | 109 | 10.3 | 2.6 | 3.2 | 4.5 | 8.6 | 2.4 | 2.7 | 3.1 | 1.8 | 0.4 | 0.5 | 0.9 |
| | 6-17y | 45 | 11.2 | 1.2 | 5.1 | 5.0 | 9.0 | 1.1 | 4.2 | 3.2 | 2.6 | 0.3 | 1.1 | 1.2 |
| | 18+y | 74 | 12.2 | 0.6 | 2.6 | 9.0 | 9.2 | 0.4 | 1.9 | 6.5 | 2.5 | 0.2 | 0.5 | 1.9 |
| | Caregiver | 154 | 11.2 | 0.8 | 2.4 | 8.0 | 8.7 | 0.7 | 1.9 | 5.6 | 2.3 | 0.2 | 0.5 | 1.6 |
| Well | Patient | 228 | 11.2 | 1.8 | 3.7 | 5.7 | 9.3 | 1.6 | 3.1 | 4.2 | 2.6 | 0.4 | 0.7 | 1.5 |
| | 0-5y | 109 | 10.5 | 2.7 | 4.0 | 3.8 | 9.2 | 2.6 | 3.7 | 2.7 | 2.3 | 0.6 | 0.8 | 0.9 |
| | 6-17y | 45 | 11.0 | 1.8 | 5.9 | 3.3 | 9.0 | 1.4 | 4.9 | 2.5 | 2.4 | 0.4 | 1.1 | 0.9 |
| | 18+y | 74 | 12.4 | 0.5 | 1.8 | 10.0 | 9.5 | 0.4 | 1.2 | 7.5 | 3.2 | 0.1 | 0.4 | 2.7 |
| | Caregiver | 154 | 11.3 | 0.7 | 2.3 | 8.3 | 9.2 | 0.6 | 2.0 | 6.1 | 2.2 | 0.2 | 0.5 | 1.5 |

**Figure 2**. Matrices of mean number of contacts when ill (the day after the clinic visit) and when well (two weeks later) by age group and contact type.

**ALL CONTACTS**

| | | All | | | | Duration>=10min | | | | Indoor | | | |
|---|---|---|---|---|---|---|---|---|---|---|---|---|---|
| | N | All | 0-5y | 6-17y | 18+y | All | 0-5y | 6-17y | 18+y | All | 0-5y | 6-17y | 18+y |
| Patient | 228 | 0.4 | 0.2 | 0.4 | -0.2 | 0.8 | 0.2 | 0.5 | 0.1 | 0.8 | 0.2 | 0.2 | 0.4 |
| 0-5y | 109 | 0.6 | 0.2 | 1.0 | -0.7 | 1.0 | 0.3 | 1.1 | -0.3 | 0.7 | 0.3 | 0.4 | 0.0 |
| 6-17y | 45 | 0.3 | 0.7 | 0.7 | -1.1 | 0.6 | 0.4 | 0.6 | -0.2 | 0.4 | 0.2 | 0.0 | 0.2 |
| 18+y | 74 | 0.3 | 0.0 | -0.8 | 1.0 | 0.5 | 0.1 | -0.5 | 1.0 | 1.0 | 0.0 | 0.0 | 1.0 |
| Caregiver | 154 | 0.3 | -0.1 | -0.1 | 0.5 | 0.7 | -0.1 | 0.1 | 0.5 | 0.1 | 0.0 | 0.1 | -0.1 |

**HOUSEHOLD CONTACTS**

| | | All | | | | Duration>=10min | | | | Indoor | | | |
|---|---|---|---|---|---|---|---|---|---|---|---|---|---|
| | | All | 0-5y | 6-17y | 18+y | All | 0-5y | 6-17y | 18+y | All | 0-5y | 6-17y | 18+y |
| Patient | 228 | 0.3 | 0.1 | 0.1 | 0.1 | 0.3 | 0.1 | 0.1 | 0.2 | 0.4 | 0.1 | 0.1 | 0.2 |
| 0-5y | 109 | 0.3 | 0.1 | 0.2 | 0.0 | 0.3 | 0.1 | 0.2 | 0.1 | 0.3 | 0.1 | 0.2 | 0.0 |
| 6-17y | 45 | 0.6 | 0.2 | -0.1 | 0.5 | 0.5 | 0.2 | -0.1 | 0.5 | 0.6 | 0.2 | -0.1 | 0.5 |
| 18+y | 74 | 0.1 | 0.0 | 0.1 | 0.0 | 0.2 | 0.0 | 0.1 | 0.1 | 0.3 | 0.1 | 0.1 | 0.2 |
| Caregiver | 154 | 0.1 | 0.0 | 0.0 | 0.1 | 0.2 | 0.0 | 0.0 | 0.1 | 0.2 | 0.0 | 0.1 | 0.1 |

**NON_HOUSEHOLD CONTACTS**

| | | All | | | | Duration>=10min | | | | Indoor | | | |
|---|---|---|---|---|---|---|---|---|---|---|---|---|---|
| | | All | 0-5y | 6-17y | 18+y | All | 0-5y | 6-17y | 18+y | All | 0-5y | 6-17y | 18+y |
| Patient | 228 | 0.1 | 0.1 | 0.3 | -0.3 | 0.4 | 0.1 | 0.4 | 0.0 | 0.4 | 0.1 | 0.1 | 0.2 |
| 0-5y | 109 | 0.2 | 0.1 | 0.8 | -0.7 | 0.7 | 0.2 | 1.0 | -0.4 | 0.4 | 0.2 | 0.2 | 0.0 |
| 6-17y | 45 | -0.2 | 0.5 | 0.8 | -1.6 | 0.0 | 0.3 | 0.7 | -0.7 | -0.2 | 0.1 | 0.1 | -0.3 |
| 18+y | 74 | 0.2 | 0.0 | -0.8 | 1.0 | 0.3 | 0.0 | -0.7 | 0.9 | 0.7 | 0.0 | -0.1 | 0.8 |
| Caregiver | 154 | 0.1 | -0.2 | -0.1 | 0.4 | 0.5 | -0.1 | 0.1 | 0.4 | -0.1 | 0.0 | 0.0 | -0.2 |

**NON-HOUSEHOLD CONTACTS AT HOME**

| | | All | | | | Duration>=10min | | | | Indoor | | | |
|---|---|---|---|---|---|---|---|---|---|---|---|---|---|
| | | All | 0-5y | 6-17y | 18+y | All | 0-5y | 6-17y | 18+y | All | 0-5y | 6-17y | 18+y |
| Patient | 228 | -0.6 | 0.1 | 0.2 | -0.8 | -0.3 | 0.1 | 0.3 | -0.5 | 0.0 | 0.0 | 0.1 | 0.0 |
| 0-5y | 109 | 0.8 | 0.2 | 0.9 | -0.2 | 0.9 | 0.2 | 0.8 | -0.2 | 0.5 | 0.1 | 0.3 | 0.1 |
| 6-17y | 45 | -2.0 | 0.0 | -0.4 | -1.5 | -1.3 | -0.1 | -0.2 | -0.7 | -0.4 | -0.1 | -0.2 | -0.1 |
| 18+y | 74 | -1.8 | 0.0 | -0.5 | -1.3 | -1.3 | 0.0 | -0.3 | -0.9 | -0.4 | -0.1 | -0.1 | -0.2 |
| Caregiver | 154 | 0.1 | 0.0 | 0.3 | -0.2 | 0.3 | 0.1 | 0.3 | 0.0 | 0.0 | 0.0 | 0.0 | 0.0 |

**NON-HOUSEHOLD CONTACTS NOT AT HOME**

| | | All | | | | Duration>=10min | | | | Indoor | | | |
|---|---|---|---|---|---|---|---|---|---|---|---|---|---|
| | | All | 0-5y | 6-17y | 18+y | All | 0-5y | 6-17y | 18+y | All | 0-5y | 6-17y | 18+y |
| Patient | 228 | 0.7 | 0.1 | 0.1 | 0.5 | 0.7 | 0.1 | 0.1 | 0.5 | 0.4 | 0.1 | 0.0 | 0.2 |
| 0-5y | 109 | -0.6 | -0.1 | 0.0 | -0.5 | -0.2 | 0.0 | 0.1 | -0.2 | 0.0 | 0.1 | 0.0 | -0.1 |
| 6-17y | 45 | 1.7 | 0.6 | 1.3 | -0.1 | 1.3 | 0.4 | 0.9 | 0.1 | 0.2 | 0.2 | 0.2 | -0.2 |
| 18+y | 74 | 1.9 | 0.0 | -0.3 | 2.3 | 1.6 | 0.0 | -0.3 | 1.8 | 1.1 | 0.0 | 0.0 | 1.0 |
| Caregiver | 154 | 0.0 | -0.2 | -0.4 | 0.6 | 0.2 | -0.1 | -0.2 | 0.5 | -0.1 | 0.0 | 0.0 | -0.1 |

Mean number of contacts higher when ill (at least 0.2 higher)
Mean number of contacts lower when ill (at least 0.2 lower)

**Figure 3**. Matrices of mean difference in number of household and non-household contacts when well compared to when ill (the day after the clinic visit) by age group and setting. Restricted to those seen on the same day of the week at each visit.
